# Medicinal plants used in traditional medicine to enhance immunity: A survey in the southeastern area of Morocco and a literature review 

**DOI:** 10.22038/AJP.2024.24096

**Published:** 2024

**Authors:** Karima EL-yagoubi, Meryem Bakour, Badiaa Lyoussi

**Affiliations:** 1 *Laboratory of Natural Substances, Pharmacology, Environment, Modeling, Health, and Quality of Life (SNAMOPEQ), Faculty of Sciences Dhar El Mahraz, University Sidi Mohamed Ben Abdellah, Fez, 30000, Morocco*; 2 *Ministry of Health and Social Protection, The Higher Institute of Nursing Professions and Health Techniques (ISPITS), Fez, 30000, Morocco*

**Keywords:** Phytotherapy, Aromatherapy, Ethnopharmacological study, Immune system

## Abstract

**Objective:**

The threat of immune escape and the discovery of antibiotic-resistant pathogens, as well as the failure of certain conventional drugs that are already in use for the symptomatic treatment of diseases, have prompted a re-evaluation of ancient therapeutic remedies such as phytotherapy, and aromatherapy. In this context, the present study aims to shed light on some medicinal plants mostly used by the population of the Daraa Tafilalet region to strengthen their immune systems, and to provide an up-to-date literature review on this subject.

**Materials and Methods:**

A survey study was conducted using pre-prepared questionnaires addressed to the population of the Daraa Tafilalet region. MS Windows Excel and SPSS software were used for statistical analysis and results presentation. In addition, for the literature review, searches were conducted across several databases, including Google Scholar, Science Direct, Web of Science, PubMed, and Scopus, using medicinal plants, date products, enhance immunity, and essential oils as keywords.

**Results:**

Among 142 participants interviewed, 108 used medicinal plants to improve their immune systems. However, 34 interviewers prefer conventional medicine. Date palm fruits and oregano were the most commonly used medicinal plants by the Daraa Tafilalet population to enhance the immune system, followed by anise, lavender, rosemary, thyme, and pennyroyal. These results are supported by literature data.

**Conclusion:**

These results reflect the interesting traditional medical knowledge of this population, which merits being documented.

## Introduction

The immune system (IS) plays a key role in the protection of the human body against aggressive environmental agents on the one hand and the harmful changes that may occur inside the organism on the other. It is a complex network of organs, cells, proteins, and chemical substances that collaborate to maintain the well-being of the organism.

Nevertheless, in some cases, the IS may not work adequately. This is because of a variety of reasons, which include but are not limited to environmental stress, infectious pathogens, and an unhealthy lifestyle that makes the body vulnerable to many diseases. Natural products are typically believed to be safer (Anywar et al., 2020). Besides, natural products with preventive and curative properties are considered direct (have immunomodulatory active compounds) or indirect (help in the management of disorders, hence maintaining a healthy environment for the IS) IS function boosters.

Moreover, many people across the world use traditional medicine; more than 80% of the world’s population utilizes alternative and complementary medicine (Nilashi et al., 2020) to protect their bodies against aggressive agents and to manage their diseases. Traditional phytotherapy is the most famous and widely used traditional and alternative healthcare approach in the world. Medicinal and aromatic plants were documented as being good for health and supporting the IS. Supplementation of healthy volunteers with 300-milligram capsules of hydroethanolic extract of *Ocimum sanctum Linn.* (Tulsi) for 4 weeks showed an interesting immunomodulatory effect. The stimulation of whole blood sample cultures isolated from Tulsi extract-supplemented volunteers with lipopolysaccaride (LPS) and phytohaemagglutinin (PHA) induces a significant increase in the levels of interferon-gamma (INF-γ), Interleukin-4 (IL-4), T helper cells, and natural killer (NK) cells as compared to whole blood cultures isolated from placebo-supplemented participants (Mondal et al., 2011).

Furthermore, many previous studies (Eddouks et al., 2020, 2017, 2002; El Rhaffari et al., 2002) affirmed that the Tafilalet population (southeastern Morocco) has shown fascinating traditional medicinal knowledge. To this end, this study aimed to investigate the medicinal plants mostly used by the population of the Daraa Tafilalet region to strengthen their IS.

## Materials and Methods

### Methodology of the review study

The literature review was carried out based on the research in several databases, including Google Scholar, Science Direct, Web of Science, PubMed, and Scopus, using the keywords "date products," "medicinal plants," "enhance immunity," and "essential oils."

### Study area

The present ethnobotanical survey was carried out in the Daraa Tafilalet region, which is located in the southeast of Morocco and covers 88.836 Km^2^ (12.5 %) of the national territory. Besides, this region contains a population of 1 635 008 habitant, which represents 4.8% of the national population (Fattahi et al., 2023).

This region is limited in the north by two regions (Fez-Meknes and Beni Mellal-Khenifra), in the east by the Oriental region, and in the west by two regions (Marrakech-Safi and Souss-Massa). The climate is semi-arid, and the temperature oscillates between -0.5°C (winter) and 42°C (summer), while the rainfall is low, generally less than 100 ml per year.

### Ethnobotanical survey

The sampling frame for this research consisted of 142 persons of both sexes from the five provinces of the Daraa Tafilalet region (Ouarzazate, Zagora, Tinghir, Midelt, and Errachidia).

Data were collected using pre-prepared questionnaires (Sphinx software), which were translated into the national language (Darija) and included a series of questions on local products used by the population of Daraa Tafilalet to improve their IS. The survey was conducted in June 2021 using two modes (direct and indirect) based on the preferences of the participants after they were told about the study's objectives. In the direct mode (which was frequently preferred by uneducated participants); one of the investigators asks the participants by using the traditional paper and pencil method (15 min per person). Instead, in the indirect mode, surveys were electronically completed by participants themselves, using their personal computers (8 min per person). The questionnaire was composed of two parts: the first one concerns socio-demographic data such as the city of origin, gender, educational level, age, and professional activity. While the second part concerns questions about the traditional remedies used by the population to strengthen their IS.

Before the interview, all participants verbally provided their informed consent. The respondents were made aware that their information would only be used for scientific research and not for any commercial purposes, and that their identities would remain confidential.

After data collection, the literature was scoured for supporting citations on the traditionally used products of interest.

### Statistical analysis

Data were entered as codes in MS Windows Excel and then processed using the Statistical Package for the Social Sciences "SPSS" version 25. The data obtained were analyzed using descriptive statistics and are presented as percentages. 

## Results

### Sociodemographic data of the present survey participants

**Figure 1:**
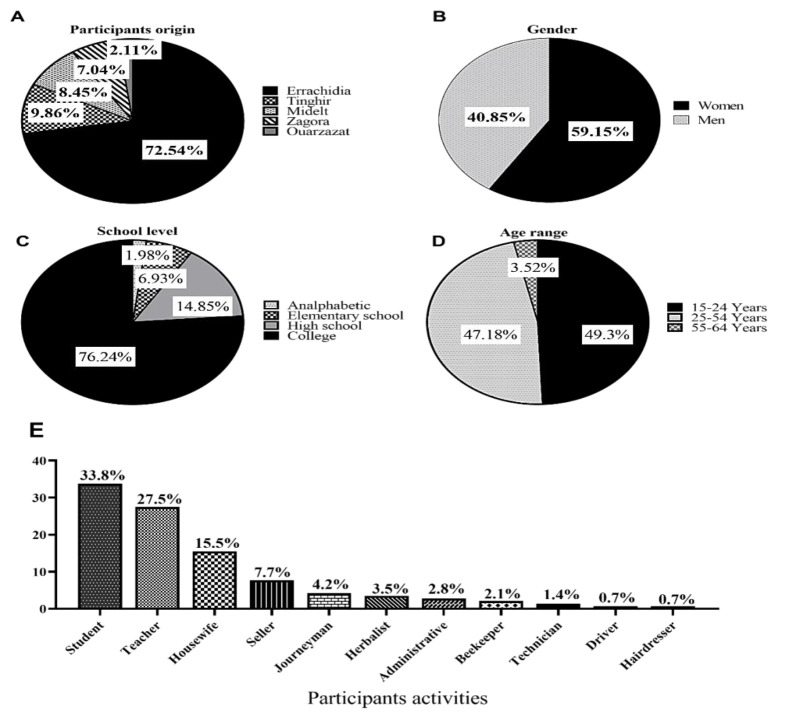
Sociodemographic data of participants

One hundred and forty-two people accepted to participate and respond to the present study questions. The majority of participants were women; they represented 59.15% of participants, while men represented 40.85%. Concerning the age range, 49.3% of participants were between 15 and 24 years old, 47.18% were between 25 and 54 years old, and 3.52% were between 55 and 64 years old. Moreover, the majority of respondents were educated: 76.24% had a university education, 14.85% had a high school education, and 6.93% had a primary school education; meanwhile, 1.98% were unschooled. Regarding the participants' origin, people from Errachidia province represented 72.54% of the present survey participants, followed by people from Tinghir, Midelt, Zagora, and Ouarzazate provinces, who represented 9.86, 8.45, 7.04, and 2.11% of the present study respondents, respectively. Concerning participant activities, 33.8% of participants were students, 27.5% were teachers, 15.5% were housewives, 7.7% were sellers, 4.2% were journeymen, 3.5% were herbalists, 2.8% were administrative, 2.1% were beekeepers, 1.4% were technicians, 0.7% were drivers, and 0.7% were hairdressers (Figure 1).

### Local products used by the Daraa Tafilalet region's population to improve their IS

Among the 142 participants, 76.1% claimed that they use aromatic and medicinal plants to improve their IS function. However, 23.9% of participants indicated that they do not prefer to use traditional products either for the treatment of their diseases or to support their IS function. 

Date palm fruits were cited as the most frequently used medicinal plants by the Daraa Tafilalet population to support their IS, followed by oregano, anise, lavender, rosemary, eucalyptus, thyme, and pennyroyal (Table 1).

Concerning the method of use of the cited plants, the date palm fruits were indicated to be consumed either as they are or in the form of date syrup or date vinegar. For the other plants, infusion, fumigation, and essential oil were the most cited forms of use. Pulp, aerial part, and seeds were reported to be the most commonly used parts of the plants. 

In addition to using the cited medicinal plants as IS function enhancing substances, it has been emphasized that they are also used for treating a range of diseases such as respiratory tract infections, gastrointestinal tract disorders, vaginal infections, and anemia. 

## Discussion

Despite the considerable advances made by modern medicine, many people around the world still use traditional medicine; it has been reported that more than 80% of the world’s population still uses complementary and alternative medicine (Nilashi et al., 2020). Similarly, this inquiry found that among the 142 participants interviewed, 76.1 % use medicinal plants to support their IS function. However, 23.9 % do not believe in traditional remedies and prefer to use conventional medicine in case of need.

Date palm fruits were cited to be the most commonly used medicinal plants, followed by oregano, anise, lavender, rosemary, eucalyptus, thyme, and pennyroyal. Their number of citations was 96.8, 90, 53, 52, 51, 36.5, 32, and 4% respectively (Table 1). Methods of use of each cited plant will be detailed in the rest of the present article.

**Table 1:** Medicinal plants used as immune-enhancer in the Daraa Tafilalet region

Used plant	Scientific name	Family	Local name	Percentage of citation	Parts used	Mode of use to enhance immunity	Other medicinal uses
Date palm fruits	*Phoenix Datylifera *L	Areacacea	Tamr	96.8%	Pulp of date fruit	Consumption of date fruit as it is, date syrup, date vinegar.	Energetic,and anemia
Oregano	*Origanum vulgare *L	Lamiaceae	Zaatar, Sahtar	90%	Arial part	Infusion, essential oil, fumigation.	Meteorism, indigestion
Anise	*Pimpinella anisum *L	Apiaceae	Habbat Hlawa	53%	Seeds	Infusion	Gastrointestinal pain
Rosemary	*Rosmarinus officinalis* L	Lamiaceae	Azir, Iklil al-jabal	52%	Arial part	Infusion, essential oil, fumigation.	Cold, vaginal infection, respiratory infection, and menstrual pain
Lavender	*Lavandula officinalis *L	Lamiaceae	L-khozama,	51%	Arial part	Infusion, essential oil, fumigation.	Hair care, vaginal infection, and respiratory tract infection
Eucalyptus	*Eucalyptus globulus *Labill	Myrtaceae	Kalitus, Kaliptus	36.5%	Arial part	Infusion, essential oil, fumigation.	Cold, and respiratory tract infection
Thyme	*Thymus vulgaris *L	Lamiaceae	Zaitra, tazoknit, Zokni	32%	Arial part	Infusion, essential oil, fumigation.	Abdominal pain
Pennyroyal	*Mentha pulegium *L	Lamiaceae	Fliou	4%	Arial part	Infusion	Cold and cough

**Table 2:** A review of the chemical composition and medicinal properties of medicinal plants used by Daraa- Tafilalet population to improve immunity

**Type of products**		**products**	**Preparation method**	**Types of study**	**Testing subjects**	**Concentration used**	**Effects**	**Chemical ** **composition**	**Reference**
Medicinal plants		Oregano	Hydroethanolic extract	*In vitro*	Human monocyte derived dendritic cells, type 1 and type 2 macrophages infected with M.bovis Bacille Calmette-Guérin (BCG)	15 µl at the concentration of (3 mg/ml)	-Enhanced the cells mycobactericidal activity. -Induced phagosome acidification and phagosomal ROS production. -Decreased TNF-α and IL-12 release. -Promoted TGF-β synthesis	-	De Santis et al., 2019
		Lavender	Hot water extraction	*In vivo*	*Cyprinus* *carpio*	0, 0.5, 1, and 1.5 % lavender extract	-Increase blood leukocytes, plasma globulin, and total Ig counts -Enhance the plasma lysozymes and alternative complement activities -Increase IL-10 and TGF-β levels -Decrease IL-1β and INF-γ levels -Enhance CAT and SOD activity -Decrease glucose and cortisol plasmatic levels	-	(Yousefi et al., 2020)
		Anise	Anise seeds decoction	*In vivo* And *In vitro*	BALB/c mice	1.5 mg in 250 ml of boiled water	-Increase blood granulocytes level and lymphocytes activity -Increase lymphocyte proliferative activity. -Reduced NO production by LPS-activated maacrophages	-	(Al-Omari et al., 2018)
Medicinal plants	Rosemary	Rosemary leaves powder	*In vivo*	*E.coli *infected cobber broiler	5 mg of rosemary leaves powder per Kg of the diet	–Increase body wheight and body wheight gain. -Increase IgG. -Decreased IL-6. -Induced partial histological improvement of organs degenerative changes induced by *E.coli *infection.	-	(Farouk et al., 2022)	
	Pennyroyal	Polyphenolic extract	*In vivo*	Male mice	15 mg/Kg	-Reduced the expression of iNOS and COX-2 -Reduced the markers of colon inflammation	Rosmarinic acid, ellagic acid, eriodictyol, naringenin, chlorogenic acid	(Rocha et al., 2019)	
	Eucalyptus	Eucalyptus leaves powder	*In vivo*	one-day-old-male chicks	0.25% and 0.5% of eucalyptus leaves powder	-Increased the WBC levels -Increase antibody production (against SRBC)	-	(Mashayekhi et al., 2018)	
Date palm products	Dates	Hot water extract	*In vivo*	Five-week-old male C3H/HeN mice	10% of mice diet	-Significantly increased the number of peyer’s patches INF-γ^+^CD4^+^, INF-γ^+^CD49^+^, and IL12^+^CD11b^+^. -Increased the spleen’s INF-γ^+^CD4^+^ and IL12^+^CD11b^+.^	Protocatechuic acid, Chlorogenic acid, Caffeic acid, Syringic acid, pelargonin, and frolic acid	(Karasawa et al., 2011)	
Essential oils	Rose and lavender essential oils	----	*In vivo*	Male Wistar rats	2% of essence (rose or lavender essentials oils) in eucerin	-Reduced the wound healing duration -Decreased the wound ulcer area from the 7^th^ day of treatment -Adequate epithelization, reduced inflammatory cell infiltration, well-formed granulation tissue of epidermis, and induced angiogenesis -Reduced formalin paw edema in rats	-	(Momtaz et al., 2021)	
	Lavender oil	Steam distillation of lavender flower heads	*In vitro*	*S. aureus* infected macrophages	-Dilution of 1/50 000 for 1×10^6^ cells	-Increased the phagocytic rate. -Reduced the intracellular replication of *S. aureus.* -Increased CYBB and NCF-4 expression. -Downregulate the expression of pro-inflammatory cytokines genes (IL-1α, IL-1β, and IL-6). -Upregulate the expression of heme oxygene-1 gene.	1,8-cineole, β ocimene, camphor, terpinene-4-ol, bergamol/linalool acetate, caryophyllene, β-linalool, borneol, and lavandulol acetate.	(Giovannini et al., 2016)	
Essential oils	Rosemary essential oil	-	*In vitro*	*S. pyogenes* *S. agalactiae* *S. pneumoniae* *K. pneumoniae* *S. aureus* *S. maltophilia*	-	-Showed antibacterial activity against tested bacteria	-	(Fabio et al., 2007)	
	Eucalyptus essential oil	Hydro-distillation of fresh and air-dry eucalyptus leaves	*In vitro*	*K. pneumonia* *S. aureus* *M. catarrhalis*	- A series of dilutions started from 50 µl of essential oil (10 mg/ml)	-Showed antibacterial activity against tested bacteria (MIC and MBC values ranged from 0.31-2.5 mg/ml and 0.63-˃5 mg/ml, respectively) -Caused cytosolic LDH release, which ranges from 8 to 24 % in comparison with TrixonX-100 -Increased rhodamine 6G accumulation in bacterial cells	-Eucalyptus fresh leaves essential oil analysis showed the presence of 31 compounds with the abundant of α-pinene, ρ-cymene, and 1,8-cineole, while dried leaves essential oil was shown to be high in 1,8- cineole, limonene, and α-pinene.	(Soyingbe et al.,2015)	
	Thyme essential oil	Prepared by a pharmacy company	Clinical trial	Patients with COVID-19	-5 ml every eight hours for seven days	-Significantly reduced the severity of COVID-19 infection symptoms, such as fever, cough, dyspnea, dizziness, muscular pain, anorexia, weakness, and fatigue. -Increased lymphocytes and calcium levels -Decreased neutrophils and blood urea nitrogen.	-	(Sardari et al., 2021)	
Essential oils	Oregano essential oil	Commercial Oreganum essential oils of tree subspecies: *Origanum compactum *(OcEO), *Origanum vulgar* (OvEO), and *Origanum vulgar* Var. htum(OhEO)	In vitro	*P. aeruginosa* isolated from cystic fibrosis patients	Oregano essential oil in concentration two-fold diluted from 2.00% to 0.06% (v/v)	-Showed antimicrobial activity even at low concentrations: 0.5% (v/v) OhEO and OvEO inhibited 80% of *P. aeruginosa* strains. While OcEO at the same concentration inhibited 65% of these bacteria-tested strains. -At concentrations lower than 0.1% (v/v), Oregano essential oils killed at least 75% of *P. aeruginosa* strains.	carvacrol (73.18%, 71.8%, 47.1%), thymol (2.30%, 1.60%, 21.5%), ρ-cymene (7.4%, 11.6%,10.8%) and γ-terpinene (3.1%, 1.7%, 8.4%) for OhEO, OvEO, and OcEO, respectively	(Maggini et al., 2017)	
	Thyme essential oil	Commercial thyme essential oil	In vitro	*S. aureus* *E. coli* *En.durans* *En.faecium* *En.faecalis * *Van B* *P. aeruginosa*	0.03125-2.5 µl/ml	-Inhibited the growth of more than 40% of *S.aureus* resistant strains at 0.5 µl/ml concentration -Inhibited the growth of 11 and 17 *Enterococcus* strains at 0.5 and 0.75 µg/ml concentrations, respectively. -At 1.25 µg/ml it inhibited the *En.faecalis* -Inhibited 13 and 17 *E .coli* strains’ growth at a concentration of 0.25 and 0.5 µg/ml, respectively. -Inhibited *P. aeruginosa* growth at 1.5- 2.5 µl/ml concentrations	Thyme essential oil analysis showed the presence of 40 compounds with the abundant of thymol, ρ-cymene, γ-terpinene, linalool, β-caryophyllene, and carvacrol	(Sienkiewicz et al., 2012)	

### Date (Phoenix dactylifera L)

Date palm trees (*Phoenix dactylifera *L) are perennial fruit trees belonging to the genus *Phoenix* in the Arecaceae family. They have been cultivated from ancient times as a staple food in the oasis of the Arab world (Khallouki et al., 2018). Besides their dietary effect, they were also used for medicinal purposes, mainly for the treatment of hypertension, diabetes, headache, dry cough, and loss of appetite (Hussain et al., 2020).

In the present survey, the majority of respondents indicated that date palm fruits are a necessary product in the Daraa Tafilalet population’s diet. It was cited as being used for its dietary and therapeutic effects, especially for its immunostimulant properties. Literary data support this traditional knowledge. Karasawa et al. ( 2011) showed that the supplementation of healthy mice with date fruit extract enhances the cellular IS more strongly than prune and fig extracts. It induced a significant increase in the number of peyer's patches interferon gamma (INF-γ^+^CD4^+^), INF-γ^+^CD49b^+^, and IL-12^+^CD11b^+^ cells as compared to non-supplemented mice. Besides, it increased the spleen’s INF-γ^+^CD4^+^and IL-12^+^CD11b^+^ cell numbers (Table 2). CD11 was documented to be the cluster of differentiation of macrophages and dendritic cells. While, CD49 was found to be the typical cell surface antigen of NK cells. Phenolic acids and β-D-glucan were suggested to be responsible for the immunostimulatory effect of date fruit. Similarly, Puri et al. (2000) found that feeding BALB/c mice with ethanolic extract of dry dates for seven consecutive days increased both humoral and cell immunity. Furthermore, date palm fruits were found to induce an immunomodulatory effect to stop the development of breast cancer. Elhemeidy et al. (2018) showed that among 7,12-dimethylbenz(a)anthracene (DMBA)-induced breast cancer rats, the level of NK cells was decreased, while that of tumor necrosis factor-alpha (TNF-α) was increased. Meanwhile, the supplementation of DMBA-induced breast cancer rats with Ajwa date extract induced a significant increase of NK cells while normalizing the level of TNF-α as compared to the non-treated group. It has been documented that, NK cells and TNF-α are key elements of anti-tumor innate immune response. However, a high level of TNF-α potentially promotes cellular transformation, cancer growth, and invasion. Moreover, date supplementation was found to normalize the breast tissue and improved the survival rates of DMBA-induced breast cancer rats. It has been documented that the health benefits of date palm fruits are attributed to their dietary fiber content (β-glucan, cellulose, pectin, tannins, and cellulose), polyphenolic compounds (flavonoids, phenolic acids, anthocyanins, and procyanidins), carotenoids, and sterols (Fernández-López et al., 2022). β-glucan is a natural molecule in date palm fruits with multiple therapeutic effects, mainly immunostimulatory properties and reduction of vulnerability to infection and cancer. The observed immunomodulatory effects of β-glucan were attributed to its ability to recognize and bind to pattern recognition receptors (PRRs) present on immune cells like monocytes, macrophages, NK cells, and neutrophils, causing a cascade of immune response that can enhance the IS protecting effect against infection and tumor proliferation (Murphy et al., 2010; Murphy et al., 2020). Additionally, Vinson et al. (2005) showed that dates had the highest content of phenolic compounds when compared to apricots, cranberries, figs, green grapes, and plums. Identification of phenolic compounds of dates varieties from Morocco showed that gallic acid is the major phenolic acid of dates, followed by ferulic acid, caffeic acid, syringic acid, p-coumaric acid, chlorogenic acid, and vanillic acid. Whereas, rutin was found to be the major flavonoid compound, followed by luteolin, and quercetin (Bouhlali et al., 2018). Gallic acid was found to improve the immune suppressive effect of anti-cancer drugs. Pretreatment of immunocompromised mice with 100 mg/kg of gallic acid increased the antibody titer, white blood cell level, and thymus weight as compared to non-treated immunocompromised groups**. **However, gallic acid in a high dose was found to be a prooxidant agent (Shruthi et al., 2018). Similarly, ferulic, caffeic, syringic, p-coumaric, chlorogenic, and vanillic acids as well as rutin, luteolin, and quercetin all have been shown to exhibit immunomodulatory effects, which may explain the immunomodulatory properties of date palm fruits (Kilani-Jaziri et al., 2017; Yahfoufi et al., 2018). It has been documented that the antioxidant activity, as well as the modulation of the inflammatory response, are common mechanisms by which polyphenols maintain the IS homeostasis (Yahfoufi et al., 2018). 

### Oregano (Origanum vulgaer L)

Oregano (*Origanum vulgare *L) belongs to the genus *Origanum* in the Lamiaceae (Labiatae) family, and it represents a famous herb used in Mediterranean cuisine. It is also known as European oregano, common oregano, and wild marjoram, but in Morocco is communally called Zaatar or Sahtar. Besides, it has been used for centuries in traditional medicine to treat asthma, diarrhea, cramping, and indigestion (Singletary, 2010).

Quantitative and qualitative analysis of oregano shows that oregano is rich in bioactive compounds. It contains volatile and non-volatile bioactive compounds. The main volatile compounds detected in oregano are carvacrol, and thymol, followed by γ-terpinene, *p*-cymene, geraniol, linalyl acetate, linalool, and *β*-myrcene. Besides, non-volatile phenolic compounds are presented by rosmarinic acid, free and glycosylated flavonoids. While luteolin is the most common flavone present in oregano followed by apigenin (Soltani et al., 2021). 

In the present survey, oregano was mostly cited to support the IS function by the Daraa Tafilalet region population. It was indicated as an IS function booster under infectious cases, mainly respiratory tract infections. Oregano leaves were mentioned to be the most oregano plant-used part. They were reported to be used in the form of oregano leaves infusion, fumigation, or oregano essential oil, either alone or mixed with honey or other plants. 

The respiratory tract represents a crucial entry point for pathogens, such as allergens, viruses, and bacteria that can potentially cause infections and diseases and then challenge the well-functioning of the IS (Lehtoranta et al., 2020). In a previous study conducted by Hassanien and Abdel-Aziz (2021), it has been found that oregano oil nano-emulsion shows an antibacterial effect on multi-antibiotics-resistant *Streptococcus* species isolated from patients with respiratory infections. The observed antibacterial effect was increased with concentration. Oregano essential oil exhibits its antibacterial effect via the alteration of the bacteria membrane integrity, which causes a cascade of events responsible for bacteria death, such as defects in the intracellular osmotic pressure, RNA synthesis, inhibition of biofilm production, and ATP loss (Hassanien and Abdel-Aziz, 2021). Carvacrol and thymol were found to be responsible for oregano oil's antibacterial effect (Fimbres-García et al., 2022; Soltani et al., 2021).

Additionally, oregano shows immunomodulatory activity which may support the host immune system against infections. It activates the intracellular microbicidal machinery and prevents potential tissue damage following an inflammatory reaction. The treatment of *Mycobacterium bovis* Bacille Calmette-Guérin (BCG)-infected peripheral blood mononuclear cells (PBMCs), namely dendritic cells, macrophage type 1 and type 2, with 15 µl of oregano ethanolic extract at the concentration of 3 mg/ml reduced the intracellular mycobacterial viability in all tested cell types. It induced phagosome acidification and reactive oxygen species (ROS) production in the tested cells and then enhanced their antibacterial defense. Whereas the ethanolic extract of oregano did not induce a direct antimicrobial effect on *M. bovis* BCG. Besides, it reduced the BCG-induced pro-inflammatory mediators' release, namely TNF-α, and IL-12, and promoted TFG-β synthesis (De Santis et al., 2019) (Table 2). Similarly, Feng and Jia (2014) showed that pretreatment with carvacrol improves lung injury in mice exposed to lipopolysaccharide (LPS) endotoxin. It has been documented that LPS endotoxin induces massive infiltration of inflammatory cells in the airway, leading to an excessive release of inflammatory mediators and then severe lung injury and respiratory failure (Feng and Jia, 2014). Pretreatment with carvacrol showed an anti-inflammatory effect via the reduction of the number of total cells, neutrophils, macrophages, TNF-α, IL-6, and IL-1β in the bronchoalveolar lavage of LPS-endotoxin-exposed mice. The carvacrol anti-inflammatory effect was related to its inhibitory effect on NF-kB and MAPK signal pathways. 

Prolonged stressor conditions influence negatively the IS function. They perturbed the normal activation and functioning of leukocytes, immunoglobulins, and inflammatory mediators (Nieto et al., 2018). Dietary supplementation of pigs with 0.2% of oregano essential oil for 190 days helped to tolerate stress-induced IS dysfunction. Peripheral blood mononuclear cells (PBMCs) isolated from pigs that were located under stress conditions showed upregulation of inflammatory response genes, including *NF-kB1, HSP90, STAT3, TNF-α, *and* IL-1β.* However, dietary supplementation of stressed pigs with oregano essential oil-induced downregulation of *NF-kB1*, *HSP90*, and *STAT3* (Nieto et al., 2018). 

### Anise (Pimpinella anisum L)

Anise (*Pimpinella anisum *L*) *also called Habbat Hlawa in Morocco, belongs to the genus *Pimpinella *in the Apiaceae family. It is a famous herb used in Moroccan cuisine. Besides, anise has long been used in traditional medicine as an analgesic, diuretic, antidiabetic, and carminative therapy (Sun et al., 2019). In our survey, anise infusion, either alone or mixed with one to two teaspoons of honey, was recommended to be good therapy to enhance the IS function. Literary data support this traditional knowledge. Mahmood et al. (2014) reported that anise has long been used as an immunostimulant remedy by many countries around the world. In a similar vein, it has been shown that daily consumption of aniseeds decoction instead of drinking water for two weeks induces a significant immunomodulatory effect in BALB/c mice (Al-Omari et al., 2018). Aniseeds-treated group showed a significant increase in blood granulocyte level and lymphocyte activity as compared to the non-treated group. Moreover, the *in vitro *phytohaemagglutinin challenge of splenocytes isolated from aniseed-treated mice significantly increased lymphocyte proliferative activity. These results support the anise stimulatory effect of cellular immunity. Besides, it showed to reduce nitric oxide (NO) production by LPS-stimulated peritoneal macrophages isolated from aniseeds-treated mice. It has been documented that NO plays a crucial inhibitory role against pathogens' multiplication. However, the overproduction of NO potentially causes uncontrolled inflammatory reactions and may be non-controlled immune responses (Al-Omari et al., 2018). Additionally, the supplementation of broiler chicks with a herbal mixture formed by 6 mg of ginger and 3 mg of aniseeds in one liter of water significantly stimulated the humoral immune response of broilers, it increased the antibody titers against newcastle diseases (ND), infectious bursal diseases (IBD), and infectious bronchitis (IB) (Raziq et al., 2023). Aniseeds analysis showed that they contain a considerable amount of essential oil. Trans-anethole, and eugenol were found to be the main compounds of this oil. Besides fatty acids-rich oil, namely palmitic and petroselinic acids. In addition to polyphenolic compounds, naringin represents the main phenolic compounds of aniseeds, followed by chlorogenic acid, quercetin, gallic, and rosmarinic acids. Anethole and eugenol were documented to be responsible for aniseeds' immunomodulatory effect (Al-Omari et al., 2018). 

### Lavender (Lavandula officinalis L)

Lavender (*Lavandula officinalis *L) known in Morocco by its vernacular name L-khozama, belongs to the genus *Lavandula* in the Lamiaceae family (Diass et al., 2021). In the present survey, lavender was mentioned to be efficient in supporting the IS. It was reported to help the body rapidly manage infections, mainly respiratory tract infections, vaginal infections, burns, and wound infections. Fumigation with lavender-dried-plant either alone or mixed with other plants was recommended to treat respiratory tract infections. While cleansing with lavender infusion, alone or with rosemary was also cited to be useful to get rid of vaginal infection. Besides, the application of lavender oil on an infected wound was reported to be helpful.

Previous studies demonstrate that lavender has a strong antimicrobial effect in *in vitro* conditions and also the ability to support the IS response under infection. Gismondi et al. (2021) found that lavender essential oil shows an antibiotic effect in a hospital context against *Staphylococcus* species and methicillin-resistant micro-organisms. In a similar vein, lavender essential oil showed significant anti-parasitic activity against *Trichomonas vaginalis* and *Giardia duodenalis*. *Trichomonas* primarily affects the urogenital system and is sexually transmissible, whereas *Giardia* mainly affects the gastrointestinal system (Moon et al., 2006). Besides, it has been found that treatment of *S. aureus*-infected human monocyte-derived macrophages (MDMs) with lavender essential oil increases the phagocytic activity and reduced the intracellular replication of *S. aureus *(Table 2). An increase in the expression of genes involved in producing ROS (*CYBB, *and* NCF-4*) was also detected in *S.aureus*–infected monocyte-derived macrophages (MDMs) after treatment with lavender essential. This observation suggests that lavender essential oil increases intracellular bacterial clearance by stimulating ROS production, crucial elements for phagocytic activity. However, the overproduction of ROS potentially causes uncontrolled inflammatory reactions. The treatment of MDMs with lavender essential oil exhibits a controlled inflammatory response induced by *S. aureus* via downregulation of the expression of pro-inflammatory cytokines genes (*IL-1α*, *IL-1β*, and *IL-6*) and upregulation of heme oxygene-1 gene expression, an anti-inflammatory factor that inhibits the production of NO by inducible nitric oxide synthase (iNOS) (Giovannini et al., 2016) (Table 2). The authors of this study suggest that the immunomodulatory effect of lavender essential oil may result from its whole phytocomplex. Gas chromatography analysis of lavender essential oil from Morocco showed that linalyl acetate is the major compound of the oil, followed by 1,8 cineol, camphor, borneol, α-terpineol, and α-bisabolol (Diass et al., 2021). Besides, the treatment of LPS-stimulated human monocytes (THP-1 cell line) with 0.1% of lavender essential oil reduced the LPS-induced anion superoxide generation, IL-1β production, TLR4 expression, and NF-kB activation (Huang et al., 2012). While it increased the expression of heat shock protein 70 (HSP-70), a cytoprotective protein that was found to be upregulated under stressor conditions, like pathogens infection. It has been proved that HSP-70 optimizes antigen presentation and processing and provides protection against chronic inflammation and hence, contributes to an efficient immune response (Jacquier-Sarlin et al., 1994). 

### Rosemary (Rosmarinus officinalis L)

Rosemary (*Rosmarinus officinalis *L) is a perennial shrub that belongs to the genus *Rosmarinus* in the Lamiaceae family. It is native to temperate areas in the Mediterranean region and it has long been used in traditional medicine as a carminative, expectorant, analgesic in muscle and joint pain, diuretic, and antispasmodic in renal colic (Andrade et al., 2018).

Bioactive compounds of rosemary are classed to be volatile (essential oil) and non-volatile compounds (polyphenols). The main constituents of rosemary essential oil are 1,8 cineol, followed by α-pinene, camphor, camphene, β-pinene, linalool, and borneol. However, the most common polyphenols of rosemary are rosmarinic acid, chlorogenic acid, caffeic acid, apigenin, diosmin, and luteolin (Andrade et al., 2018). 

In the present inquiry, rosemary was cited to help support the IS functioning. It was recommended to help the body get rid of infectious pathogens, mainly those attacking the vaginal and respiratory tract. Rosemary leaves and flowers were reported to be the most commonly used part and the main cited remedies were fumigation with rosemary aerial part; either alone or mixed with other plants, direct application of rosemary essential oil, consumption of rosemary aerial part infusion; either alone or mixed with honey, and cleansing with rosemary decoction.

On one hand, rosemary essential oil was found to exhibit a direct antibacterial effect in multi-drug resistant bacteria responsible for respiratory tract infection (Table 2): *Streptococcus pyogenes*, *Streptococcus agalactia*e, *Streptococcus pneumoniae*, *Klebsiella pneumoniae*, *Staphylococcus aureus*, and *Stenotrophomonas maltophilia* isolated from clinical specimens (Fabio et al., 2007). Additionally, Bogavac et al. (2017) showed that rosemary essential oil had interesting antibacterial and antifungal activities against multidrug-resistant Gram-positive and Gram-negative bacteria and two candida strains responsible for vaginal infection in pregnant women. The intensity of inhibition of microbial growth and multiplication was positively correlated to rosemary essential oil concentration and exposure duration. The possible mechanism of action of the antibacterial effect is the interaction of rosemary compounds with bacteria membrane proteins which leads to the destruction of membrane structure and function. Besides, this antibacterial activity was also suggested to be related to the alteration of electron transport, and changes in fatty acids, and genetic material of bacteria by rosemary active compounds (Nieto et al., 2018). Rosemary's antibacterial effect was related to the synergistic effect of its bioactive compounds (Bogavac et al., 2017; Nieto et al., 2018). 

On the other hand, the dietary supplementation of *E. coli*-infected cobber broiler with 5 mg of rosemary leaves powder per Kg of the diet each for 6 weeks induced a significant reduction of IL-6 and increased the level of IgG as compared to the control infected broiler. Besides, *E. coli *infection induced a significant depletion of the bursa of Fabricius and thymus lymphocytes and deleterious changes of broiler organs, namely thymus, bursa of Fabricius, kidneys, gut, and liver. However, supplementation with rosemary induced partial histological improvement (Farouk et al., 2022) (Table 2). This result highlights the possible immunomodulatory effect of rosemary under infection. Furthermore, rosemary supplementation was found to enhance the IS response against a non-pathogenic antigen: sheep red blood cells (SRBCs). Eight weeks of rosemary supplementation of BALB/c mice immunized with sheep blood cells enhanced the mice humoral response, it induced a significant increase in IgM and IgG response to SRBCs. The rosemary crude extract at 50 mg/kg was found to be the optimum dose for inducing antibody response in mice (Al Sheyab et al., 2012). Besides it has been found that oral supplementation of mice with rosemary extract increases T-cells proliferation in response to concanavalin A antigene. The dose of 100 mg/kg was found to exhibit the ideal effect of T-cells proliferation (Al Sheyab et al., 2012).

### Eucalyptus (Eucalyptus globulus Labill.)

Eucalyptus *(Eucalyptus globulus *Labill*.) *commonly called Kalitus or Kaliptus in Morocco, is a flowering plant from the genus *Eucalyptus* in the Myrtacea family. It has been long used by Australian people for the treatment of colds, coughs, congestion, joint pain, skin disorders, and insect bites (Coppen, 2002). It is the world’s primary source of eucalyptus essential oil, 1,8 cineol or eucalyptol is the main compound of the oil (Coppen, 2002). 

In our present study, people from the Daraa Tafilalet region recommended eucalyptus as an efficient remedy to support IS function during respiratory tract infections. They reported that inhalation of eucalyptus leaves decoction vapor is helpful to manage respiratory tract infection-related diseases. Eucalyptus leaves essential oil as well as fumigation with this plant leaves, either alone or mixed with other plants, were recommended to be also efficient remedies. 

In a similar vein, Soyingbe et al. (2015) tested the antimicrobial activities of eucalyptus essential oil against *Klebsiella pneumonia*, *Staphylococcus aureus*, and *Moraxella catarrhalis*, three respiratory tract infectious microorganisms. They showed that both fresh and dried eucalyptus leaf-derived essential oils exhibit antimicrobial activity against tested strains (the minimum inhibitory concentration (MIC) and minimum bactericidal concentration (MBC) of these oils ranged between 0.31 and 2.5 mg/ml and from 0.63 to more than 5 mg/ml, respectively) via destroying the bacteria’s membrane integrity as well as disrupting the bacteria’s efflux pump function (Table 2). Besides its antimicrobial effect, eucalyptus essential oil was found to modulate the phagocytic activity of mycobacterium-infected alveolar macrophages. The pre-treatment and post-treatment of infected alveolar macrophages with 0.02% of eucalyptus essential oil significantly increased the phagocytic activity as well as bacterial clearance (Yadav and Chandra, 2017). Furthermore, eucalyptus essential oil and its main compound, 1,8 cineol regulated the inflammatory response of LPS-challenged macrophages. Pretreatment of alveolar macrophage with 0.02% of eucalyptus essential oil 3 hr before LPS stimulation, reduced the secretion of NO and pro-inflammatory mediators, namely IL-1α, IL-1β, and TNF-α. However, pretreatment with 0.02% of 1,8 cineol induced a reduction of IL-1α, IL-1β, and IL-6 synthesis only. The observed anti-inflammatory effect of eucalyptus essential was correlated to the downregulation of mRNA expression of trigger receptor presents on myeloid cells 1 (TREM1) as well as the reduction of phosphorylation of p38 MAPK and NF-kB (Yadav and Chandra, 2017).

Additionally, Lin et al. (2017) showed that oral administration of eucalyptus essential oil attenuated emphysematous damage and thickness of bronchioles in a chronic obstructive pulmonary disease rat model. They also reported that eucalyptus essential oil treatment markedly reduced levels of inflammatory cells in the blood and bronchoalveolar lavage liquid and decreased proinflammatory cytokine TNF-α and IL-β release in lung homogenate. It also reduced malondialdehyde (MDA) formation and increased superoxide dismutase (SOD) activity. Moreover, it has been found that 1,8-cineol exhibits medical benefits in inflammatory airway diseases such as asthma and chronic obstructive pulmonary disease (Juergens, 2014). It has been found that 1,8-cineol improves the inflammatory status in the airways by direct inhibition of mucus hypersecretion stimulatory mediators, mainly prostaglandins (prostaglandin (PG2) and thromboxane B2 (TxB2)), leukotrienes (LTB4), and ROS. 1,8-cineol was documented to be a competitive histamine receptor antagonist (Juergens, 2014). 

### Thyme (Thymus vulgaris L)

Thyme (*Thymus vulgaris *L) also called garden thyme or common thyme is a member of the genus *Thymus* in the Lamiaceae family. It is originating from the Mediterranean area and it has been used for many centuries as a culinary, cosmetic, and therapeutic product. It has been long used for the treatment of a wide range of diseases which include but are not limited to urinary infections, vaginal irritation, wound healing, respiratory infections, *H-pylori*, and intestinal parasites (Basch et al., 2004). 

It has been found that hydrodistillation of dried thyme yields 1-2.5% essential oil. Thymol is the main volatile compound of thyme essential oil, followed by carvacrol, an isomer of thymol, γ-terpinene, and *p*-cymene two precursors of thymol and carvacrol. Additionally, linalool, borneol, camphor, myrcene, *ß*-pinene, trans-sabinene hydrate, α-terpineol, and terpinene-4-ol were also found in a smaller percentage (Stahl-Biskup and Venskutonis, 2012). Besides, thyme contains non-volatile phenolic compounds, namely rosmarinic acid, caffeic acid, gentisic acid, *p*-cumaric acid, syringic acid, ferulic acid, and *p*-hydroxybenzoic acid as well as free and glycosylated flavonoids (Stahl-Biskup and Venskutonis, 2012). 

In the present study, thyme was also cited to be used to support the IS response against environmental pathogens, namely those responsible for respiratory tract infections. Leaves were mentioned to be the main used part, and they are recommended to be taken in the form of an infusion; either alone or mixed with honey, fumigation, or essential oil.

In a similar vein, one-week treatment of COVID-19 patients with thyme essential oil significantly reduced the severity of COVID-19 infection symptoms, which include but are not limited to fever, cough, dyspnea, dizziness, muscular pain, anorexia, weakness, and fatigue. Besides, blood urea nitrogen (BUN) and neutrophil count were also decreased. However, calcium and lymphocytes were increased (Sardari et al., 2021) (Table 2). Furthermore, Maggini et al. (2017) and Sienkiewicz et al. (2012) showed that thyme and oregano essential oils considerably inhibit the growth of multidrug-resistant bacteria isolated from patients, clinical staff, and hospital environments. It was suggested that thymol, carvacrol, γ-terpinene, and ρ-cymene might synergistically contribute to the antibacterial activity. Besides, Kumari et al. (2017) evaluated the antifungal efficacity of six active compounds of Lamiaceae essential oil against *Cryptococcus neoformans* and *Cryptococcus laurentii* the main causative agents of systemic mycosis which mainly affects immunocompromised patients. Among the six tested compounds (thymol, carvacrol, eugenol, citral, cinnamaldehyde, and menthol), thymol and carvacrol were found to have the best antifungal effect. They exhibit the lower minimum inhibitory concentration as compared to the other tested compounds, which inhibits 80% of *Cryptococcus neoformans* and *Cryptococcus laurentii* growth (MIC_80%_), biofilm formation (BIC_80%_), and performed biofilm (BIC_80%_). 

Besides its direct effect on harmful pathogens which may perturb the well-being of the human body, thyme and its active compounds were proven to exhibit an immunomodulatory effect which helps strengthen the well-functioning of the immune system. The treatment of hepatic coccidiosis-infected rabbits with thyme essential oil at a dose of 500 mg/kg completely stopped the oocytes shedding at day 34 post-infection with *Eimeria stiedae* oocysts as compared to moringa oil (on day 41). The result showed that thyme oil has a strong anticoccidial effect and can eliminate infection earlier than moringa oil. Besides, thyme increased the IgG response from the first week of treatment. However, at the end of the experiment, the IgG level of thyme-treated group was lower than that observed in the moringa and non-treated group. The observed effect was suggested to be related to the earlier elimination of infection with thyme, which was manifested at the end by a decrease in IgG antibodies titer (Abu El Ezz et al., 2020). Similarly, the pretreatment of LPS-stimulated mammary epithelial cells of mice (mMECs) with thymol reduced the LPS-induced proinflammatory cytokines release, namely IL-6, and TNF-α in a dose-dependent manner. Besides, it induced inhibition of iNOS and cyclooxygenase-2 (COX-2) expression. The anti-inflammatory effect of thymol occurred via the inhibition of NF-kB and MAPK pathways (Liang et al., 2014). Furthermore, Gholijani and Amirghofran (2016) showed that incubation of splenocytes isolated from ovalbumin (OVA)-immunized mice significantly increased these cells' proliferation and IL-2, INF-γ, IL-4, IL-17, and IL-23 cytokines production. However, it reduced IL-10 and transforming growth factor (TGF-β) synthesis. Whereas, treatment of mice with thymol and carvacrol reduced the *ex-vivo* proliferation of splenocytes and IL-2, INF-γ, IL-4, IL-17, and IL-23 cytokines production. In contrast, they increased IL-10 and TGF-β synthesis. It has been documented that (IL-2, and INF-γ), (IL-4), (IL-17, and IL-23), and (IL-10) are key cytokines of Th1, Th2, Th17, and Treg cells respectively. An imbalance in the cells' subsets activity leads to IS-related disorders (Gholijani and Amirghofran, 2016).

### Pennyroyal (Mentha pulegium L.) 

Pennyroyal (*Mentha pulegium *L.) commonly called Fliyou in Morocco, belongs to the *Mentha* genus in the Lamiaceae family. In the present survey, pennyroyal was cited to be used to support the IS function, particularly during winter. Pennyroyal dried leaves infusion, either in hot water or hot milk was reported to be the most commonly used remedy. Besides, it can be consumed alone or mixed with one to two teaspoons of honey. Similarly, Sheikhian et al. (2016) reported that pennyroyal is used by Iranian people to enhance their IS function. It has been found that pennyroyal could enhance the IS of healthy animal models. The daily peritoneal injection of healthy mice with hydroethanolic extract of pennyroyal at a dose of 200 mg/kg for twenty days induced a significant increase in reed and weight blood cells as well as the hemoglobin concentration (Modaresi and Iranpour, 2014). However, pennyroyal was found to support the IS function under pathological conditions, like inflammatory diseases. Rocha et al. (2019) showed that the treatment of trinitrobenzenesulfonic acid (TNBS)-induced colitis mice with pennyroyal phenolic extract induced a significant reduction of colon inflammatory markers: intestinal inflammatory injury, diarrhea severity, and expression of inflammatory markers. The anti-inflammatory effect of pennyroyal phenolic extract was mediated by the inhibition of COX-2 and iNOS protein expression (Table 2). In a similar vein, the treatment of LPS-stimulated RAW 274.6 cells with pulegone, a predominant compound of pennyroyal essential oil, induced a significant inhibition of LPS-induced NO overproduction in a dose-dependent manner. It has been found that pulegone exhibits its anti-inflammatory effect via the downregulation of NF-kB and MAPK signaling pathways, and the upregulation of the anti-inflammatory HO-1/Nrf2 signaling pathway (Roy et al., 2018). Despite the showed immunomodulatory effect of pulegone, prior studies have found that it is a toxic compound and its use should be reasonable (Hadi et al., 2017). 

In conclusion, the present ethnobotanical study highlighted the main aromatic and medicinal plants used by Daraa Tafilalet population to support their bodies' IS function. These results reflect the richness of the Daraa Tafilalet region in products with interesting therapeutic uses, in addition to the existing traditional medical knowledge of this population, which merits being documented. However, even if the mentioned products, namely the prepared remedies, show health improvement after their traditional use, they should be used cautiously. For this reason, future studies are needed to identify the active compounds and the possible pharmacological activities of the mentioned remedies (synergistic or cumulative effects), their therapeutic and toxic doses, and the appropriate routes of administration.
